# Lifestyle and environmental factors in women carrying *BRCA* pathogenic variants with and without cancer

**DOI:** 10.1093/jncics/pkaf097

**Published:** 2025-10-14

**Authors:** Alessandra Fabi, Antonio Franco, Laura Cortesi, Emanuela Lucci Cordisco, Alessandro Rossi, Daniela Andreina Terribile, Elena Fiorio, Ida Paris, Giovanni Terrana, Edoardo Mocini, Vanda Salutari, Francesco Pavese, Francesca Federica L’Erario, Domizia Pasquetti, Antonella Palazzo, Enrico Di Guglielmo, Alejandro Martin Sanchez, Sabatino D’Archi, Elena Giontella, Alba Di Leone, Ludovica Cardinali, Rosa Saltarelli, Lorenzo Scardina, Elisabetta Ferretti, Luisa Carbognin, Michele Milella, Carlo Baldari, Roberto Bei, Antonella Carcagni, Giorgia Garganese, Gianluca Franceschini, Maurizio Genuardi, Diana Giannarelli, Silvia Migliaccio

**Affiliations:** Precision Medicine Unit in Senology, Fondazione Policlinico Universitario A. Gemelli IRCCS, Rome, Italy; Breast Unit, Fondazione Policlinico Universitario A. Gemelli IRCCS, Rome, Italy; Division of Oncology, Department of Oncology and Hematology, University Hospital of Modena, Modena, Italy; Medical Genetics, Fondazione Policlinico Universitario A. Gemelli IRCCS, Rome, Italy; Department of Life Sciences and Public Health, Università Cattolica del Sacro Cuore, Rome, Italy; Precision Medicine Unit in Senology, Fondazione Policlinico Universitario A. Gemelli IRCCS, Rome, Italy; Breast Unit, Fondazione Policlinico Universitario A. Gemelli IRCCS, Rome, Italy; Innovation Biomedicine—Oncology Area, Department of Engineering for Innovation Medicine, University of Verona and Verona University and Hospital Trust, Verona, Italy; Division of Gynecologic Oncology, Department of Woman and Child Health and Public Health, Fondazione Policlinico Universitario A. Gemelli IRCCS, Rome, Italy; Department of Experimental Medicine, University Sapienza of Rome, Rome, Italy; Department of Theoretical and Applied Sciences, eCampus University, Novedrate, Italy; Division of Gynecologic Oncology, Department of Woman and Child Health and Public Health, Fondazione Policlinico Universitario A. Gemelli IRCCS, Rome, Italy; Division of Gynecologic Oncology, Department of Woman and Child Health and Public Health, Fondazione Policlinico Universitario A. Gemelli IRCCS, Rome, Italy; Medical Genetics, Fondazione Policlinico Universitario A. Gemelli IRCCS, Rome, Italy; Medical Genetics, Fondazione Policlinico Universitario A. Gemelli IRCCS, Rome, Italy; Medical Oncology, Fondazione Policlinico Universitario A. Gemelli IRCCS, Rome, Italy; Breast Unit, Fondazione Policlinico Universitario A. Gemelli IRCCS, Rome, Italy; Breast Unit, Fondazione Policlinico Universitario A. Gemelli IRCCS, Rome, Italy; Breast Unit, Fondazione Policlinico Universitario A. Gemelli IRCCS, Rome, Italy; Innovation Biomedicine—Oncology Area, Department of Engineering for Innovation Medicine, University of Verona and Verona University and Hospital Trust, Verona, Italy; Breast Unit, Fondazione Policlinico Universitario A. Gemelli IRCCS, Rome, Italy; Department of Life Science, Health, and Health Professions, Link Campus University, Rome, Italy; Complex Operative Unit of Oncology, San Giovanni Evangelista Hospital, Tivoli, Italy; Breast Unit, Fondazione Policlinico Universitario A. Gemelli IRCCS, Rome, Italy; Department of Experimental Medicine, University Sapienza of Rome, Rome, Italy; Precision Medicine Unit in Senology, Fondazione Policlinico Universitario A. Gemelli IRCCS, Rome, Italy; Innovation Biomedicine—Oncology Area, Department of Engineering for Innovation Medicine, University of Verona and Verona University and Hospital Trust, Verona, Italy; Department of Theoretical and Applied Sciences, eCampus University, Novedrate, Italy; Department of Clinical Sciences and Translational Medicine, Tor Vergata University, Rome, Italy; Epidemiology and Biostatistica, Fondazione Policlinico Universitario A. Gemelli IRCCS, Rome, Italy; Breast Unit, Fondazione Policlinico Universitario A. Gemelli IRCCS, Rome, Italy; Breast Unit, Fondazione Policlinico Universitario A. Gemelli IRCCS, Rome, Italy; Medical Genetics, Fondazione Policlinico Universitario A. Gemelli IRCCS, Rome, Italy; Department of Life Sciences and Public Health, Università Cattolica del Sacro Cuore, Rome, Italy; Epidemiology and Biostatistica, Fondazione Policlinico Universitario A. Gemelli IRCCS, Rome, Italy; Department of Experimental Medicine, University Sapienza of Rome, Rome, Italy

## Abstract

**Background:**

In the development of breast cancer and ovarian cancer there may be an influence of lifestyle and environmental factors. This influence could be relevant also in patients with genetic predisposition such as in carriers of germline pathogenic variants in the *BRCA* genes. However, this issue has been addressed in only a few studies so far.

**Methods:**

In this retrospective, multicenter case-control study, we enrolled participants with a pathogenic variant *BRCA* gene and divided into 2 groups: group 1, patients with breast cancer and/or ovarian cancer, and group 2, subjects without cancer. We compared these groups regarding demographic data as age, body mass index, smoking habits, estroprogestinic use, Mediterranean diet, and physical activity. Multivariable analyses were used to identify predisposing factors. All evaluations were 2-tailed and considered statistically significant if the *P* value was less than .05.

**Results:**

We enrolled 281 participants, 135 (79.4%) with breast cancer, 32 (18.8%) with ovarian cancer, 3 (1.8%) with both, and 111 unaffected (39.5%) women. Independent risk factors associated with cancer were age (*P *< .0001); body mass index (*P *= .007); family history (*P *= .002); occupation (*P *= .003); smoking habits (*P *= .012), number of cigarettes smoked (*P *= .016), and pack-year index (*P *= .022); and estroprogestinic use (*P *= .032) and years of estroprogestinic use (*P *= .029). At multivariate analysis, age (odds ratio [OR] = 1.062; *P *< .0001), family history (OR = 0.129; *P *= .001), number of cigarettes smoked (*P *= .014), and estroprogestinic use (OR = 2.009; *P *= .025) were statistically significant risk factors associated with cancer development.

**Conclusions:**

In the development of breast cancer and ovarian cancer, lifestyle and environmental factors seem to play a statistically significant role in the presence of genetic predisposition associated with *BRCA1* and *BRCA2* gene mutations.

## Introduction

Breast cancer is the most frequent cancer in women; the lifetime probability of developing invasive breast cancer is estimated to be 12.8%. Ovarian cancer represents the second most common type of gynecologic cancer, with an incidence of 1.5%.^1^ Their incidence appears to be higher in Europe and North America and lower in Africa and Southeast Asia in relation to different habits and lifestyle.^2^^,^^3^ A sedentary lifestyle and being overweight seem to play a role in the development of both cancer types. Indeed, it has been described for 2 decades that an increase in body mass index (BMI) of 5 units is correlated with a 12% and 7%-10% increased risk of developing breast cancer and ovarian cancer, respectively.^4-6^

Additionally, genetic factors are also related to the pathogenesis of breast cancer and ovarian cancer. In approximately 20% of patients with triple-negative breast cancer and 20%-25% of women with high-grade serous ovarian cancer, a germline *BRCA* pathogenetic variant gene (*gBRCA* VP) can be found. The *BRCA* genes are involved in homologous recombination repair of double-strand DNA breaks. *BRCA* pathogenetic variant genes disrupt gene function and cause genomic instability, favoring tumor development.^7^ Individuals carrying *BRCA1* pathogenetic variant genes have a cumulative risk of developing breast cancer of 72% (95% confidence interval [CI] = 65% to 79%) and 69% in case of *BRCA2* genes (95% CI = 61% to 77%). Regarding ovarian cancer, the risk associated with the presence of a *BRCA* pathogenetic variant gene in the case of *BRCA1* and *BRCA2* is 44% (95% CI = 36% to 53%) and 17% (95% CI = 11% to 25%), respectively. This risk estimate is higher in women who carry the *BRCA* pathogenetic variant gene who have multiple relatives with breast cancer. A similar association with family history was observed for the risk of ovarian cancer.^8-12^

Further, it is known that the exposome plays an important role in the development of cancer.^8^ Exposure to many environmental toxic factors, such as bisphenol A and cadmium, has been demonstrated to play a role in the development of cancer acting as an endocrine disruptor and thus, leading to hormonal carcinogenesis.^13^ For instance cadmium, a proven carcinogen absorbed into the body from dietary sources, cigarette smoke, and by inhalation in industrial or polluted environments, binds to and activates the estrogen receptor in breast cancer cells promoting cell proliferation through the activation of several different pathways including *PI3K-NRF2*.^13-16^ These observations are consistent with the hypothesis that genetic risk related to cancer development might be modified by environmental factors. Previous studies have evaluated factors such as lifestyle, smoking habits, estroprogestinic use, overweight, weight gain, and physical inactivity as potential additional risk elements of breast cancer or ovarian cancer in *BRCA* pathogenetic variant gene carriers with inconclusive results.^17-19^ The aim of the STILVARCA study is to evaluate the possible influence of environmental and lifestyle factors in the development of breast cancer or ovarian cancer in *BRCA* pathogenetic variant gene carriers.

## Methods

This is a retrospective, multicenter case-control study (NCT05748353) involving 5 participating centers conducted from January to September 2022. Females with a genetic test diagnostic of a *BRCA* pathogenetic variant gene performed between January 2012 and December 2021 were recruited. The enrolled population was divided into 2 groups: group 1, women with breast cancer and/or ovarian cancer; group 2, unaffected women.

Inclusion criteria were women aged older than 18 years with no upper limit; signature of informed consent; histologic diagnosis of breast cancer (stage I-III with any histotype) or ovarian cancer (stage I-III and any histotype); presence of *BRCA* pathogenetic variant genes or likely pathogenic variants (class V or IV)^20^; no previous prophylactic mastectomy and/or bilateral ovariectomy. Exclusion criteria were absence of variants of *BRCA1* or *BRCA2* class IV or V pathogenic variants, patients who underwent prophylactic breast or ovarian surgery, and patients with stage IV breast cancer or ovarian cancer.

Data collection was prospectively updated in a unique database. This study was conducted in accordance with the ethical standards as laid down in the Declaration of Helsinki and was approved by the central ethics committee of Fondazione Policlinico Agostino Gemelli, Rome (number: RS 4472).^21^

### Data collection

Data collected were acquired before the diagnosis of cancer for group 1 and at enrollment for group 2 and are shown in Table S1. The difference in environmental exposure assessment is intended to prevent the change in the patient’s lifestyle and habits following the diagnosis of malignancy from invalidating the study results. The following datasets were collected:

1. Demographic: age (at cancer diagnosis for group 1 and at enrollment for group 2); BMI; family history (presence of other first grade family members with breast cancer or ovarian cancer); ethnic group; gene involved (*BRCA1* or *BRCA2*); occupation (divided into employed, educative professions and students, health-care professions, unemployed or retired, and other)
2. Smoking habits divided into no smoker or ex-smoker, smoker of less than 10 cigarettes per day, smoker of 10-20 cigarettes per day, and smoker of more than 20 cigarettes per day; years of smoking; pack-year index
3. Estroprogestin exposure including use and duration of estroprogestinic use
4. Cancer description: site of cancer (breast cancer or ovarian cancer); surgery (demolitive or conservative for breast cancer and demolitive for ovarian cancer)
5. Histotype
6. Biological subtypes for breast cancer (luminal tumor [estrogen receptor and/or progesterone receptor positivity, HER2 negative [0, 1+, 2+; with silver in situ hybridization or fluorescence in situ hybridization not amplified]; triple-negative [estrogen and progesterone receptors negative, HER2 negative]; HER2-enriched estrogen receptor and progesterone receptor negative, HER2 2+ silver in situ hybridization/fluorescence in situ hybridization amplified or HER2 3+])
7. Oncological treatments (neoadjuvant chemotherapy [NACT] or adjuvant chemotherapy)
8. Nutritional evaluation with adherence to Mediterranean diet (reported by PREDIMED questionnaire) (Figure S1A)^22^
9. Physical activity evaluated with the International Physical Activity Questionnaire short form (IPAQ-SF) (Figure S1B)

The PREDIMED questionnaire, a validated 17-item tool, assesses adherence to the Mediterranean diet. Scores are categorized as follows: poor adherence (≤7), moderate adherence,^8-10^ and high adherence (≥11). The IPAQ-SF questionnaire measures the type and amount (days per week and minutes per day) of physical activity performed during the week considering 3 types of activities: vigorous, moderate, and walking. Total metabolic activity equivalents (METs) were calculated as the sum of walking, moderate, and vigorous MET scores. Physical activity was classified as moderate when there were at least 3 days of vigorous intensity activity lasting at least 20 minutes per day; or at least 5 days of moderate intensity activity and/or walking for at least 30 minutes per day; or at least 5 days of any combination of walking, moderate intensity, or vigorous intensity activities with a total of at least 600 MET minutes per week. The high category comprised vigorous intensity activity on at least 3 days at a total of at least 1500 MET minutes per week; or 7 or more days of any combination of walking, moderate intensity, or vigorous intensity activities at a total of 3000 or more MET minutes per week. Individuals not meeting the criteria for moderate or vigorous categories were classified as low physical activity. Questionnaires were submitted during the outpatient visit.

### Statistical analyses

Continuous variables were described by mean (SD) (median and interquartile range) and compared with Student *t* test. Categorical variables have been described by absolute number and percentage, and associations were assessed with the χ^2^ test. Initially, we evaluated differences of these factors between group 1 and group 2. Then, we tested these aspects on the different types of neoplasia by identifying a group of patients with breast cancer, a group of patients with ovarian cancer, and a group of unaffected patients. Finally, we performed univariate and multivariable analyses, using binary logistic regression, aiming to identify predisposing factors for cancer development. Odds ratios (ORs) were reported along with their 95% confidence intervals. All statistical evaluations were 2-tailed and considered significant if the *P-*value was less than .05 (*P *< .05). Statistical analysis was performed using SPSS ver. 26.0 (Statistical Package of Social Sciences).

## Results

We enrolled 281 patients (Table 1). Of these patients, 170 (60.5%) had a previous cancer: 135 (79.4%) breast cancer, 32 (18.8%) ovarian cancer, and 3 (1.8%) both breast cancer and ovarian cancer. The remaining 111 (39.5%) patients had no history of cancer.

**Table 1.** Characteristics of patients with cancer diagnosis (group 1)

Breast cancer	Total patients, No. (%)
	(*n* = 135)
Biological subtype	
Triple negative	61 (45.2)
Luminal B	44 (32.6)
Luminal A	15 (11.1)
HER2 enriched^a^	15 (11.1)
Histotype	
Invasive ductal carcinoma	117 (86.7)
Invasive lobular carcinoma	11 (8.1)
Invasive carcinoma no special type	7 (5.2)
Systemic treatment	
Neoadjuvant CT	54 (40.0)
Adjuvant CT	42 (31.1)
No CT	39 (28.9)
Surgery	
Conservative^b^	43 (31.9)
Demolitive^c^	92 (68.1)

Ovarian cancer	Total patients, No. (%)
	(*n* = 32)
Histotype	
High-grade serous carcinoma	28 (87.5)
Endometrioid ovarian carcinoma	2 (6.25)
Clear cell ovarian carcinoma	2 (6.25)
Systemic treatment	
Adjuvant CT	24 (75.0)
No CT	8 (25.0)
Surgery	
Radical hysterectomy with annessiectomy, lymphadenectomy and omentectomy, and selective peritonectomy	18 (56.3)
Radical hysterectomy with annessiectomy	8 (25.0)
Radical hysterectomy with annessiectomy and lymphadenectomy/omentectomy	6 (18.7)

Breast and ovarian cancer	Total patients, No. (%)
	(*n* = 3)
Histotype	
High-grade serous carcinoma plus invasive ductal carcinoma	3 (100.0)
Treatment	
Neoadjuvant CT	3 (100.0)
Surgery	
Demolitive breast surgery^c^ plus radical hysterectomy with annessiectomy, lymphadenectomy/omentectomy, and selective peritonectomy	3 (100.0)

Abbreviation: CT = chemotherapy.

a HER2 2+ silver in situ hybridization and fluorescence in situ hybridization amplified, HER2 3+.

b Quadrantectomy or monolateral conservative or demolitive mastectomy.

c Bilateral conservative or radical mastectomies.

The 2 most common breast biological subtypes were triple negative (61 [45.2%] patients) and luminal B (44 [32.6%] patients). Among the patients, 86.7% (117 of 135) showed ductal invasive carcinoma. Most patients received chemotherapeutic treatment (54 [40%] NACT; 42 [31.1%] adjuvant chemotherapy). Only 39 (28.9%) breast cancer patients received only antihormonal treatment. Finally, complete ablative surgery (mastectomy of both breasts) was performed in 92 (68.1%) patients. Conservative surgery was chosen when patients had not yet been diagnosed with a *BRCA* pathogenetic variant gene at the time of surgery or when there were contraindications to bilateral mastectomy.

The most common histotype in ovarian cancer was high-grade serous ovarian carcinoma. This was observed in 28 (87.6%) patients. Among all patients, 18 (56.3%) underwent radical surgery (radical hysterectomy plus adnexectomy, lymphadenectomy and omentectomy, and selective peritonectomy). Most patients underwent adjuvant chemotherapy (24 [75%]).

Finally, 3 patients showed synchronous breast cancer and ovarian cancer. All showed high-grade serous ovarian carcinoma and breast invasive ductal carcinoma and underwent NACT followed by bilateral mastectomy.

Overall, the mean age was 42.8 (12.1) years (Table 2). The mean age was higher in group 1 (44.7 [10.0] years in group 1 and 37.3 [12.9] years in group 2; *P *< .0001]). BMI was statistically significant higher in group 1 (24.5 [4.4] kg/m^2^) as compared with group 2 (23.0 [4.1] kg/m^2^; *P *= .006). A total of 251 (89.3%) patients had a positive family history. Group 2 had a higher incidence of positive family history (97.3% vs 86.5%; *P *< .001). Group 1 presented a higher fraction of patients with smoking habits (38.8% vs 24.3%; *P *= .008), as well as a higher number of cigarettes smoked. Group 1 presented a higher percentage of smokers of more than 10 cigarettes per day than group 2 (23.5% vs 8.1%; *P *= .008). This is associated with a statistically higher number of index pack-years for patients with cancer (4.2 [8.1] vs 2.0 [5.8]; *P *= .001). Finally, the last difference was in estroprogestinic use. A significantly higher and longer (for more years) use of estroprogestinic use was observed in group 1: 33.5% in group 1 and 21.6% in group 2 (*P *= .021).

**Table 2.** Description and comparison of group 1 and group 2 patient characteristics

Characteristics	Total patients, No. (%)	Group 1 patients with breast cancer and/or ovarian cancer, No. (%)	Group 2 patients without breast cancer and ovarian cancer, No. (%)	*P*
	(*n* = 281)	(*n* = 170 [60.5%])	(*n* = 111 [39.5%])	
*BRCA* pathogenic variants				.804
*BRCA* 1	168 (59.8)	103 (60.6)	65 (58.6)	
*BRCA* 2	113 (40.2)	67 (39.4)	46 (41.4)	
Age, mean (SD), y	42.8 (12.1)	44.7 (10.0)	37.3 (12.9)	**.001**
Body mass index, mean (SD), kg/m^2^	23.9 (4.3)	24.5 (4.4)	23.0 (4.1)	**.006**
Family history				**<.001**
Yes	251 (89.3)	143 (84.1)	108 (97.3)	
No	30 (10.7)	27 (15.9)	3 (2.7)	
Occupation				**.001**
Employed	132 (47.0)	85 (50.0)	47 (42.3)	
Educative profession	26 (9.3)	15 (8.8)	11 (9.9)	
Health-care profession	45 (16.0)	27 (15.9)	18 (16.2)	
Unemployed or retired	46 (16.3)	34 (20.0)	12 (10.8)	
Other	32 (11.4)	9 (5.3)	23 (20.7)	
Smoking habits				**.008**
Yes	93 (33.1)	66 (38.8)	27 (24.3)	
No	188 (66.9)	104 (61.2)	84 (75.7)	
No. cigarettes per day				**.008**
Non smoker	188 (66.9)	104 (61.2)	84 (75.7)	
<10	44 (15.6)	26 (15.3)	18 (16.2)	
10-20	35 (12.5)	28 (16.5)	7 (6.3)	
>20	14 (5.0)	12 (7.0)	2 (1.8)	
Index pack-years, mean (SD)	3.3 (7.3)	4.2 (8.1)	2.0 (5.8)	**.001**
Estroprogestinic use				**.021**
Yes	81 (28.8)	57 (33.5)	24 (21.6)	
No	200 (71.2)	113 (66.5)	87 (78.4)	
Years of estroprogestinic use, mean (SD)	1.8 (4.6)	2.4 (5.1)	1.1 (3.4)	**<.0001**
Mediterranean diet				.201
Low adherence	28 (10.0)	20 (11.8)	8 (7.2)	
Moderate adherence	113 (40.2)	62 (36.5)	51 (45.9)	
High adherence	140 (49.8)	88 (51.8)	52 (46.8)	
Physical activity				.509
Inactive	86 (30.6)	51 (30.0)	35 (31.5)	
Moderate active	116 (41.3)	67 (39.4)	49 (44.1)	
Active	79 (28.1)	52 (30.6)	27 (24.3)	

Considering the factors associated with individual disease (Table S2), the factors that maintain statistical significance among the 3 groups are age (higher among ovarian cancer patients; *P *< .0001), BMI (higher in ovarian cancer patients; *P *= .010), family history (especially in ovarian cancer patients; *P *= .001), and smoking habits, which is confirmed to be more present in patients with neoplasm (*P *= .039). The association with estroprogestinic intake loses significance (*P *= .085).

In the univariate analysis (Table 3), independent risk factors associated with cancer were age (*P *< .0001); BMI (*P *= .007); family history (*P *= .002); occupation (*P *= .003); smoking habits (*P *= .012), number of cigarettes smoked (*P *= .016), and pack-year index (*P *= .022); and estroprogestinic use (*P *= .032) and years of estroprogestinic intake (*P *= .029). In a multivariable analysis, age (OR = 1.062; *P *< .0001), family history (OR = 0.129; *P *= .001), number of cigarettes smoked (*P *= .014), and estroprogestinic use (OR = 2.009; *P *= .025) were negative predictive factors associated with cancer development.

**Table 3.** Univariate and multivariable analysis for cancer development

Characteristics	Univariate analysis		Multivariable analysis	
	OR (95% CI)	*P*	OR (95% CI)	*P*
Age, y	**1.063 (1.038 to 1.088)**	**<.0001**	**1.062 (1.037 to 1.090)**	**<.0001**
Body mass index, kg/m^2^	**1.087 (1.023 to 1.154)**	**.007**		
Family history		**.002**		**.001**
No	Referent		Referent	
Yes	**0.147 (0.043 -to 0.498)**	**.002**	**0.129 (0.037 to 0.451)**	**.0001**
Occupation		**.003**		
Employed	**4.622 (1.977 to 10.802)**	**<.0001**		
Educative profession	**3.485 (1.166 to 10.418)**	**.025**		
Health-care profession	**3.833 (1.447 to 10.157)**	**.007**		
Unemployed or retired	**7.241 (2.628 to 19.948)**	**<.0001**		
Other	Referent			
Smoking habits		**.012**		
No	Referent			
Yes	**1.974 (1.160 to 3.361)**	**.012**		
No. cigarettes per day		**.016**		**.014**
Non smoker	Referent		Referent	
<10	1.167 (0.599 to 2.271)	.650	1.146 (0.560 to 2.344)	.709
10-20	**3.231 (1.344 to 7.764)**	**.009**	**3.472 (1.386 to 8.699)**	**.008**
>20	**4.846 (1.055 to 22.253)**	**.042**	**5.440 (1.080 to 27.406)**	**.040**
Pack/years index	**1.052 (1.007 to 1.099)**	**.022**		
Estroprogestin use (EP)		**.032**		**.025**
No	Referent		Referent	
Yes	**1.829 (1.052 to 3.178)**	**.032**	**2.009 (1.092 to 3.696)**	**.025**
Years of estroprogestinic use	**1.074 (1.007 to 1.146)**	**.029**		
Mediterranean diet		.203		.118
Low adherence	1.477 (0.607 to 3.593)	.389		
Moderate adherence	0.718 (0.434 to 1.190)	.199		
High adherence				
Physical activity		.510		.441
Inactive	0.757 (0.402 to 1.425)	.388		
Moderate active	0.710 (0.392 to 1.285)	.258		
Active	Referent			

Abbreviations: CI = confidence interval; OR = odds ratio.

## Discussion

Breast cancer and ovarian cancer are 2 of the most common cancers in women all over the world. Their occurrence is associated with different risk factors. The most common causes were age, ethnic group, menarche history, breast gland characteristics, family history, reproductive patterns, pregnancy at a later age, estroprogestinic and alcohol use, smoking habits, diet, and physical activity.^23-25^ In some cases, their occurrence is linked to *BRCA* pathogenetic variant genes or other genes that cause a high risk (up to 72% for breast cancer and up to 44% for ovarian cancer).^11^^,^^26^ Increasing evidence supports that genetic predisposition is influenced by environmental factors, likely acting synergistically with genetics to develop cancer.^27^

Interestingly, several environmental factors such as bisphenol A and cadmium as well as other phthalates are well known to play a role in hormonal carcinogenesis.^28-29^ In particular, cadmium, released into the soil, water, and air from industrial processing and absorbed into the body from dietary sources, cigarette smoke, and by inhalation in industrial or polluted environments, acts as an activator of cell proliferation.^30-32^ In fact, it binds the estrogen receptor in breast cancer cells and promotes cell proliferation through the activation of *PI3K-NRF2*.^13-16^ Further, we have recently demonstrated a role of cadmium as well as other environmental pollutants in hormonal carcinogenesis of breast cancer in preclinical experimental models, demonstrating a potential pivotal role of the exposome in cancer development.^30-33^

To our knowledge, few studies have evaluated the influence of environmental factors in population carrier of *BRCA1* and *BRCA2* pathogenetic variant genes. Furthermore, few studies have investigated the role of a harmful lifestyle and obesity in the development of cancer in the same participants. Our purpose was to compare patients with genetic predisposition by dividing them into 2 groups (patients who developed breast cancer and/or ovarian cancer and patients unaffected) evaluating whether and what environmental factors had any influence in the development of cancer.

We enrolled 281 patients (168 [59.8%] *BRCA1* and 113 [40.2%] *BRCA2* carriers) into a case-control study. There was no statistically significant difference between the 2 groups according to the gene involved, with equal distribution of pathogenetic variant genes in *BRCA1* and *BRCA2*, respectively, in patients developing cancer and those unaffected at time of observation. Overall, 170 (60.5%) developed breast cancer and/or ovarian cancer and 111 (39.5%) had no diagnosis at the last observation.

These 2 groups differed regarding age at the time patients had a positive gene test and time of cancer development. Patients with cancer had a higher mean age (44.7 years vs 37.3 years; *P *< .0001). This difference can be explained because usually the first genetic test (the diagnostic test) is performed in a family when there is evidence of cancer. Subsequently, cascade genetic testing is performed by family members, often younger and healthy, to verify whether they have inherited the family-specific variant and have proper risk and surveillance advice. Subgroup analysis showed that patients with ovarian cancer have a higher age of onset of neoplasia than patients with breast cancer and without neoplasia (Table S2). The difference in age at onset could also explain the difference regarding family history. Patients that are unaffected have a higher incidence of cancer among family members (97.3% vs 84.1%; *P *< .001) because they were tested especially if a family member had the pathogenetic variant.

The 2 groups differ also regarding smoking habits, which was higher among ovarian cancer vs breast cancer patients (38.8% vs 24.3%; *P *= .008). Group 1 showed a higher number of cigarettes smoked (7% vs 1.8% participants with more than 20 cigarettes per day smoked and 16.5% vs 6.3% smoking between 10 and 20 cigarettes per day). This was associated with an higher pack-year index (4.2 vs 2.0; *P *= .001). Furthermore, we also recorded a statistically significant difference regarding the use of estroprogestin. Patients with cancer used estroprogestin more than unaffected subjects (33.5% vs 21.6%; *P *= .021) and for a longer time (2.4 vs 1.1 years; *P *< .001). However, the use of estroprogestin does not seem to affect patients with ovarian cancer.

Finally, we found that independent factors for cancer development were BMI (OR = 1.087; *P *= .007) and occupation (*P *= .003). However, we did not find a relationship between the development of cancer and both physical activity and Mediterranean diet adherence (group 1 reported poor adherence to the Mediterranean diet compared with group 2, although it did not reach statistical significance [11.8% vs 7.2%; *P *= .201]). Although BMI emerged as an independent factor for cancer development, it is worth noting that adherence to healthier dietary patterns, such as the Mediterranean diet, has been associated with lower BMI and improved metabolic profiles. Interestingly, a systematic review of the literature and a mini review investigated the role of modifiable factors on malignancy development in women who were *BRCA* mutation carriers, describing few and contradictory results.^33^^,^^34^ This not statistically significant trend could suggest that preexisting dietary habits, potentially combined with other lifestyle factors, may have contributed to cancer development in susceptible individuals. However, this hypothesis requires confirmation in larger cohorts, as postdiagnosis changes in dietary behavior may also confound these observations.

In the multivariable analysis, 4 factors were associated with a predictive role: age, family history, smoking habits, and estroprogestin use. Age was predictive (OR = 1.062; *P *< .0001) probably because of the longer exposure to environmental or lifestyle factors leading to a higher possibility of developing cancer. The second risk factor was family history, namely whether another first-degree family member had been affected by breast or ovarian cancer, as previously reported in the literature.^35^ However, healthy patients usually undergo genetic testing only if at least one family member is positive. In our study, healthier patients have at least 1 family member with cancer (OR = 0.129; *P *= .001). The third factor was smoking habits; women with smoking habits may have a higher chance of developing cancer. This possibility increases with as the number of cigarettes smoked increases (*P *= .014). This evidence could be associated with cadmium exposure, present in inhaled smoke. The greater the number of cigarettes smoked, the greater the inhalation of cadmium and the greater the induced damage to DNA and the risk of developing cancer. We confirmed, even in patients with *BRCA* pathogenetic variant genes, that smoking has a causal role in the development of breast cancer.^36^ We also obtained evidence for a potential role of estroprogestin use. The assumptions of estroprogestin use caused an increased risk of developing cancer, especially breast cancer (OR = 2.009; *P *= .025).^37^ One possible explanation for this finding is the presence of a high number of hormones that stimulate the proliferation of glandular cells, which in the presence of an altered DNA repair mechanism could promote the development of cancer. However, the use of estroprogestin does not seem to affect ovarian cancer development. Some studies report data on a protective role of estroprogestins on the occurrence of ovarian cancer.^38^

Our study is primarily limited by its retrospective design. The information was obtained before diagnosis of cancer and at enrollment for healthy patients, therefore, changes in lifestyle after treatment could have altered the results. After diagnosis, patients might have changed their lifestyle with the introduction of healthy modifications and, thus, potential alteration of responses to the questionnaire. Another weakness was the lack of data on serum cadmium levels, which could have further helped the potential direct role of this environmental contaminant as a risk factor in malignancy development.

One strength is that the inclusion of women with and without cancer enhances the robustness of the study design by allowing a direct comparison between groups, thereby improving the identification of environmental and lifestyle factors that may influence cancer development in *BRCA* pathogenetic variant gene carriers. Additionally, the inclusion of validated tools (17-item PREDIMED questionnaire for dietary adherence and IPAQ-SF for physical activity) ensures the reliability and reproducibility of the collected data. The study captures demographic, lifestyle, and clinical information, facilitating a thorough analysis of factors influencing cancer risk. Finally, we used a multidisciplinary approach. Integrating genetic, environmental, and lifestyle factors, the study adopts a holistic and multidisciplinary approach that is essential for understanding the complex interplay between genetic predisposition and environmental influences. A prospective study is also warranted to capture further information in an era of greater awareness of the population’s lifestyles.

## Supplementary Material

pkaf097_Supplementary_Data

## Data Availability

The data underlying this article are available in the article and in its online supplementary material.
