# Supplementary material for: Lifestyle and environmental factors in women carrying BRCA pathogenic variants with and without cancer
Source: JNCI Cancer Spectr. 2025 Oct 14;9(6):pkaf097. doi: 10.1093/jncics/pkaf097 (PMC12681321; doi:10.1093/jncics/pkaf097)
Supplement: pkaf097_Supplementary_Data [file pkaf097_supplementary_data.zip › JNCICS-01.07 Supplemental materials_28.06.2025.docx]

Supplemental Table

| Table S1: Patient and Tumor Characteristics Required by the Study | | |
| --- | --- | --- |
| Epidemiological Information | Age | Years |
|  | Body mass index (BMI) | Kg/m^2^ |
|  | Famiy History | Presence in family of other cancer |
|  | Ethnic group |  |
|  | BRCA gene with pathological variant | BRCA 1  BRCA 2 |
|  | Occupation | Employed  Educative professions/students  Healthcare professions  Unemployed/retired  Other occupations |
| Smoking Habits (SH) ^#^ | Smoker | Yes/No |
|  | Number of cigarettes smoked for day | No smoker/ex-smoker  Smoker < than 10 cigarettes/die  Smoker of 10-20 cigarettes/die  Smoker > than 20 cigarettes/die |
|  | Years of smoking |  |
|  | Pack/years index^a^ | Index describing the number of cigarettes smoked during lifetime |
| Previous Estro-Progestin exposure^#^ | Use of EP | Yes/no |
|  | Years of EP intake |  |
|  | In vitro Fertilization (IVF) | Yes/no |
| Tumor  Information | Site of cancer | Breast cancer (BC)  Ovarian Cancer (OC) |
|  | Type of surgery *^b^* | *Breast cancer*  Demolitive (bilateral conservative or radical mastectomy); Conservative (quadrantectomy mono- o bilateral)  *Ovarian cancer*  Radical hysterectomy with annexectomy; Radical hysterectomy with annexectomy and lymphadenectomy/omentectomy; Radical hysterectomy with annexectomy, lymphadenectomy/omentectomy and selective peritonectomy |
|  | Histological definitions | BC: invasive ductal carcinoma, invasive lobular carcinoma, invasive carcinoma no special type  Luminal A; Luminal B, Triple negative, HER2+  OC: high-grade serous ovarian carcinoma (HGSOC), clear cell ovarian carcinoma (CCOC), endometrioid (ENOC), mucinous carcinoma (MUC) |
|  | Chemotherapic (plus or not biological agents) Treatment | Neoadjuvant treatment (NACT)  Adjuvant treatment (ACT) |
| Nutritional evaluation^#^ | Adherence to Mediterranean diet | PREDIMED questionnaire (17-item) |
| Physical activity^#^ | Physical activity evaluation | IPAQ questionnaire |
| ^a^ *it is calculated by multiplying the number of packs of cigarettes smoked per day by the number of years the person has smoked*  *^b^ definitions for breast cancer. Surgery for ovarian cancer is always hystero-annexectomy with lymphadenectomy*  *^#^before the cancer diagnosis or at the time of the enrollment for patients without cancer diagnosis* | | |

| *Table S2: Comparison of patient characteristics according to presence and kind of cancer* | | | | | |
| --- | --- | --- | --- | --- | --- |
| ***Characteristics*** | ***N. (%)***  ***Total patients***  ***281*** | ***Patients with BC***  ***135 (48.0%)*** | ***Patients with OC***  ***35 (12.5%)*** | ***Patients without cancer***  ***111 (39.5%)*** | ***p-value*** |
| **Kind of BRCA VP**  - BRCA 1  - BRCA 2 | 168 (59.8%)  113 (40.2%) | 82 (60.7%)  53 (39.3%) | 21 (60.0%)  14 (40.0%) | 65 (58.6%)  46 (41.4%) | *0.970* |
| **Age (years)** | 42.8 ± 12.1 | 42.9 ± 8.9 | 51.4 ± 10.2 | 37.3 ± 12.9 | ***<0,0001*** |
| **BMI (Kg/m^2^)** | 23.9 ± 4.3 | 24.3 ± 4.3 | 25.3 ± 4.4 | 23.0 ± 4.1 | ***0.010*** |
| **Family History**  - Yes  - No | 251 (89.3%)  30 (10.7%) | 114 (84.4%)  21 (15.6%) | 29 (82.9%)  6 (17.1%) | 108 (97.3%)  3 (2.7%) | ***0.001*** |
| **Occupation**  - Employed  - Educative profession  Healthcare profession  - Unemployed/retired  - Other | 132 (47.0%)  26 (9.3%)  45 (16.0%)  46 (16.3%)  32 (11.4%) | 70 (51.9%)  12 (8.9%)  21 (15.6%)  26 (19.3%)  6 (4.4%) | 15 (42.9%)  3 (8.6%)  6 (17.1%)  8 (22.9%)  3 (8.6%) | 47 (42.3%)  11 (9.9%)  18 (16.2%)  12 (10.8%)  23 (20.7%) | ***0.011*** |
| **Smoking habits**  - Yes  -No | 93 (33.1%)  188 (66.9%) | 53 (39.3%)  82 (60.7%) | 13 (37.1%)  2 (62.9%) | 27 (24.3%)  84 (75.7%) | ***0.039*** |
| **Cigarettes/day**  - No smokers  - <10 cigarettes/die  - 10-20 cigarettes/die  - >20 cigarettes/die | 188 (66.9%)  44 (15.6%)  35 (12.5%)  14 (5.0%) | 82 (60.7%)  19 (14.1%)  23 (17.0%)  11 (8.1%) | 22 (62.9%)  7 (20.0%)  5 (14.3%)  1 (2.9%) | 84 (75.7%)  18 (16.2%)  7 (6.3%)  2 (1.8%) | ***0.026*** |
| **Index pack/years** | 3.3 ± 7.3 | 4.1 ± 8.3 | 4.6 ± 7.8 | 2.0 ± 5.8 | *0.055* |
| **EP^a^ use**  - Yes  - No | 81 (28.8%)  200 (71.2%) | 46 (34.1%)  89 (65.9%) | 11 (31.4%)  24 (68.6%) | 24 (21.6%)  87 (78.4%) | *0.085* |
| ***Years of EP*^a^ *use*** | 1.8 ± 4.6 | 2.5 ± 5.1 | 2.1 ± 5.6 | 1.1 ± 3.4 | *0.069* |
| **Mediterranean diet**  - Low adherence  - Moderate adherence  - High adherence | 28 (10.0%)  113 (40.2%)  140 (49.8%) | 19 (14.1%)  47 (34.8%)  69 (51.1%) | 1 (2.9%)  15 (45.9%)  19 (54.3%) | 8 (7.2%)  51 (45.9%)  52 (46.8%) | *0.139* |
| **Physical activity**  - Inactive  - Moderate active  - Active | 86 (30.6%)  116 (41.3%)  79 (28.1%) | 38 (28.1%)  53 (39.3%)  44 (32.6%) | 13 (37.1%)  14 (40.0%)  8 (22.9%) | 35 (31.5%)  49 (44.1%)  27 (24.3%) | *0.564* |
| **^a^** *EP: Estro-progestin* | | | | | |

**Supplemental figures**


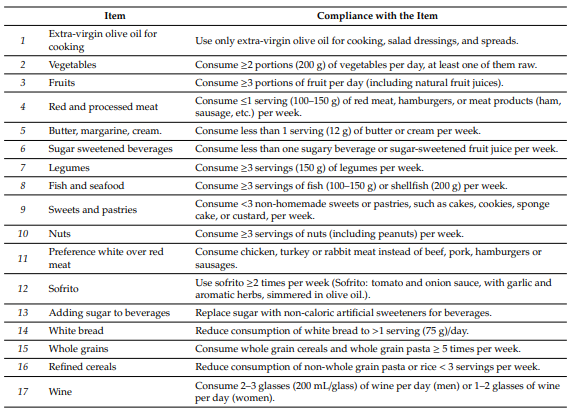


*Figure S1a: 17 item-PREDIMED questionnaires*


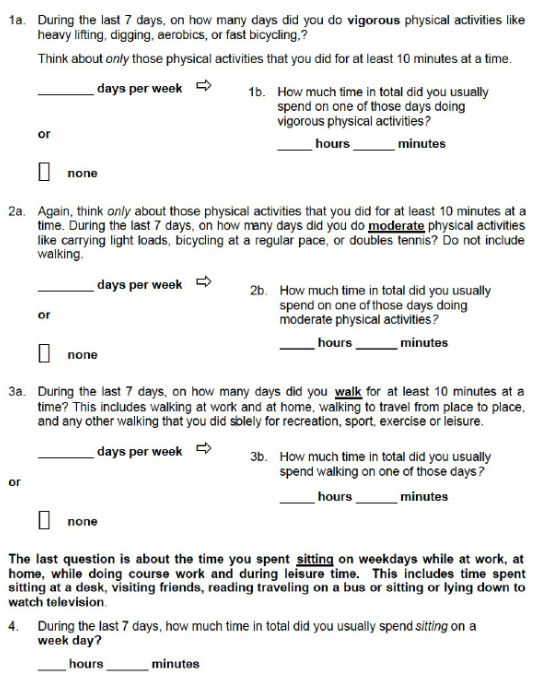


*Figure S1b: IPAQ questionnaire*
